# Biosensor Based on Electrochemical Analysis for *Staphylococcus aureus* Detection with Molecular Imprinted Polymer Technique

**DOI:** 10.3390/polym17212826

**Published:** 2025-10-23

**Authors:** Naphatsawan Vongmanee, Jindapa Nampeng, Chuchart Pintavirooj, Sarinporn Visitsattapongse

**Affiliations:** Department of Biomedical Engineering, School of Engineering, King Mongkut’s Institute of Technology Ladkrabang, Bangkok 10520, Thailand; naphatsawan.v@hotmail.com (N.V.); jindapa.na@kmitl.ac.th (J.N.); chuchart.pi@kmitl.ac.th (C.P.)

**Keywords:** *Staphylococcus aureus*, hydroxyproline, molecular imprinted polymer, electrochemical sensor, screen-printed electrode

## Abstract

*Staphylococcus aureus* (*S. aureus*) is one of the most common hospital-acquired pathogens and poses a serious threat to patients with weakened immune systems. Transmission can occur through foodborne illness, skin infections, abscess formation, and bloodstream invasion. The most severe complication arises when *S. aureus* infects the heart, leading to valve damage and potentially progressing to heart failure. In addition, many strains have developed strong resistance to conventional antibiotic therapies, making treatment increasingly difficult. These challenges highlight the importance of early detection for effective prevention and management. This research focuses on the development of a polymer composite incorporating hydroxyproline for the preparation of molecularly imprinted polymers (MIPs) designed for the rapid detection of *S. aureus*. The sensing platform, based on electrochemical principles, enabled sensitive and efficient analysis of bacterial samples. The sensor exhibited a broad analytical range, detecting *S. aureus* from 1 to 10,000 CFU/mL, with a detection limit as low as 1.031 CFU/mL. Selectivity testing against *Pseudomonas aeruginosa*, *Candida albicans*, and *Escherichia coli* confirmed high specificity toward *S. aureus*. These findings highlight the potential of this MIP-based electrochemical sensor as a reliable tool for rapid bacterial detection in clinical and environmental settings.

## 1. Introduction

*Staphylococcus aureus* (*S. aureus*) is recognized as a major human pathogen responsible for a wide range of serious illnesses and is increasingly associated with premature mortality [1]. As a Gram-positive bacterium, it functions as an opportunistic pathogen capable of causing diverse infections such as sepsis [2], pneumonia [3], urinary tract infections [4], bloodstream infections [5], wound and surgical site infections [6], meningitis [7], and hepatic abscesses [8]. The burden of *S. aureus* infections continues to rise worldwide, highlighting its significant impact on its clinical importance and the urgent demand for improved diagnostic, preventive, and therapeutic strategies [9]. Conventional laboratory methods for identifying *S. aureus* primarily depend on biochemical assays of bacterial colonies [10]. These include tests for clumping factor, coagulase activity, hemolysin production, and thermostable deoxyribonuclease [11]. While effective in routine diagnostics, these techniques are often time-consuming [12], typically requiring one to three days of incubation before results can be confirmed. In addition, the accuracy of these assays depends heavily on skilled laboratory personnel [13]. The prolonged turnaround time, operational complexity, and elevated costs associated with these methods limit their efficiency, especially in settings that require rapid diagnostics [14]. Consequently, there is a critical need for alternative detection strategies that are faster, more selective, and cost-effective [15].

Biosensors are analytical devices that couple a biological recognition element with a physicochemical transducer to detect target analytes [16]. They can be broadly classified based on the type of signal transduction used, such as optical, thermal, piezoelectric, and electrochemical biosensors [17]. Among these, electrochemical biosensors are particularly attractive because of their high sensitivity, portability, low cost, and ability to provide rapid responses [18,19,20,21,22]. Owing to these advantages, electrochemical biosensors have been extensively applied in medical diagnostics, food safety, environmental monitoring and bacterial detection [23,24]. To overcome these limitations, this study introduces an electrochemical sensing approach integrated with the molecularly imprinted polymer (MIP) technique for the rapid detection of *S. aureus*. Molecularly Imprinted Polymers (MIPs) are synthetic polymers which possess highly specific recognition sites complementary in shape, size, and functional groups to a target molecule or analyte [25]. The molecular imprinting process involves the polymerization of functional monomers and cross-linkers in the presence of a template molecule [26,27]. After polymerization, the template is removed, leaving behind cavities that can selectively rebind the target analyte [28,29]. The sensor was designed using a polymer composition composed of hydroxyproline and acrylamide as functional monomers. Hydroxyproline, a non-protein amino acid, plays a structural role in plants, animals, and human cells [30]. Importantly, in Gram-positive bacteria such as *S. aureus*, which possess smooth cell walls with thick peptidoglycan layers, hydroxyproline demonstrates the capacity to interact with collagen-like structures [31]. This property was exploited in the present design, allowing bacterial recognition through binding interactions that can be monitored via oxidation–reduction current changes measured by cyclic voltammetry [32]. Acrylamide was employed as a complementary monomer due to its extensive use in biological applications; despite its known neurotoxicity [33], it has been extensively used in biological and polymer applications [34]. Its amide group is capable of forming hydrogen-bonding and electrostatic interactions with hydroxyl, carboxyl and phosphate groups present on the surface of *S. aureus* cells [35]. These interactions facilitate the creation of stable and selective recognition cavities within the imprinted polymer. Optimization of both hydroxyproline and acrylamide ratios was necessary to achieve the most effective polymerization conditions for selective recognition of *S. aureus*. Polymerization was carried out using cross-linkers, initiators, and solvents, followed by removal of the bacterial template to generate selective recognition sites on the electrode surface [36]. Incorporation of graphene oxide (GO) into the polymer matrix provided a high surface area and enhanced conductivity, thereby facilitating efficient electron transfer and increasing the availability of binding sites [37,38]. This molecular imprinting process ensured that the electrode was capable of selectively binding *S. aureus* cells.

The resulting MIP-based electrochemical sensor demonstrated several advantages, including rapid response, high sensitivity, and ease of use for bacterial analysis [39,40]. The platform allowed detection of *S. aureus* across a wide concentration range and was validated for selectivity against other microorganisms such as *Pseudomonas aeruginosa*, *Candida albicans*, and *Escherichia coli*. These results highlight the potential of this biosensor as a promising diagnostic tool, capable of providing rapid and reliable detection of *S. aureus* in both clinical and environmental applications.

## 2. Materials and Methods

### 2.1. Staphylococcus aureus Preparation

**Figure 1:**
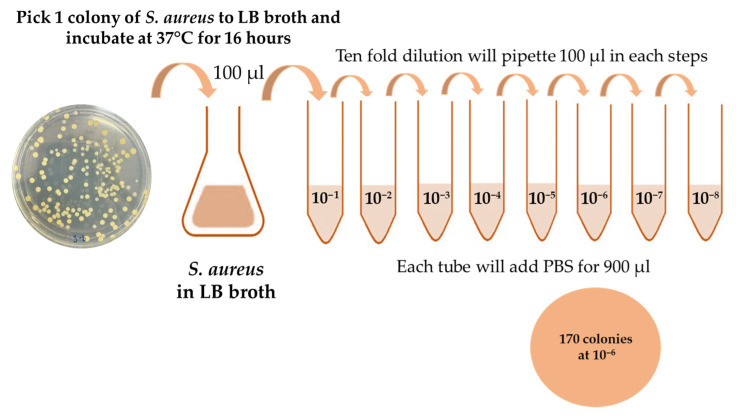
Schematic for ten-fold dilution of *S. aureus*; 170 colonies were counted after sixth dilution.

*Staphylococcus aureus* (ATCC 25923, Manassas, VA, USA) was cultured in Luria–Bertani (LB) broth and incubated at 37 °C with shaking at 240 rpm for 16 h to achieve optimal bacterial growth. Upon completion of incubation, spread plating was performed to determine colony-forming units (CFUs) and establish the initial bacterial stock concentration, as illustrated in Figure 1.

For working dilutions preparation, the bacterial culture was subjected to stepwise tenfold serial dilutions using PBS as the diluent. In the first step, 100 µL of the overnight *S. aureus* suspension was added to 900 µL of PBS, yielding a 1:10 dilution. Subsequent dilutions were prepared by transferring 100 µL of each preceding dilution into 900 µL of PBS, repeated as needed to obtain the desired concentration range. The resulting dilutions were spread plated on LB agar, and the CFU counts were recorded after incubation to calculate the initial stock concentration. These standardized dilutions were then used for subsequent experimental assays to ensure reproducibility and accuracy in bacterial quantification.

**Figure 2:**
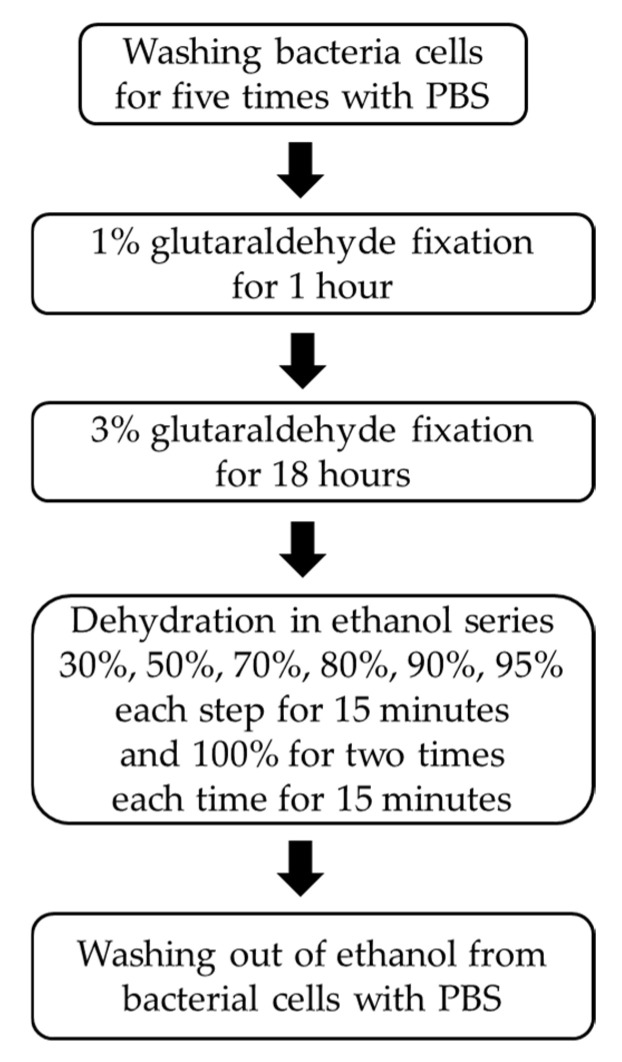
Procedure for bacteria fixation with glutaraldehyde and dehydration process with ethanol series.

Prior to imprinting, bacterial cells were fixed to preserve their morphology, including size and shape. To remove residual nutrients, the bacterial suspension was first washed with phosphate-buffered saline (PBS). The cells were then subjected to glutaraldehyde fixation, beginning with a 1% solution for 1 h, followed by immersion in a 3% solution for 18 h. After fixation, the cells were dehydrated through a graded ethanol series to ensure structural stability. The bacterial samples were sequentially soaked in ethanol solutions at 30%, 50%, 70%, 80%, 90%, and 95% concentrations, each for 15 min. Finally, two washes in 100% ethanol, each lasting 15 min, were performed to complete the dehydration process. This stepwise fixation and dehydration ensured the preservation of cellular morphology required for molecular imprinting, as illustrated in Figure 2. After dehydration, the residual ethanol was removed by washing the bacteria cells three times with PBS and the bacteria cells were finally resuspended in 1 mL of PBS.

### 2.2. Quantification of S. aureus Cells Applied to the Electrode Surface

The number of *Staphylococcus aureus* cells that could theoretically occupy the working electrode surface was estimated using the ratio between the electrode area and the average surface area of a single bacterium:

(1) $$N=\frac{A_{electrode}}{A_{cell}}$$

where *A_electrode_* is the electrode surface area and *A_cell_* is the projected surface area of a single *S. aureus* cell.

The screen-printed working electrode used in this study had a surface area of 12.56 mm^2^. Based on reported diameter, *S. aureus* cells are approximately 0.5–0.8 µm in diameter, corresponding to an average 2-D projected surface area of 0.34 µm^2^ per cell. From these values, the theoretical maximum coverage was calculated to be approximately 3.69 × 10^7^ cells per electrode. However, in practice, only about 10^5^ cells are typically required for the imprinting process. This reduced number is chosen deliberately to prevent excessive crowding and overlapping of bacterial templates on the electrode surface, which can negatively affect the imprinting quality and recognition performance of the molecularly imprinted polymer sensor.

### 2.3. Molecular Imprinted Polymer on Screen Printed Electrode

**Figure 3:**
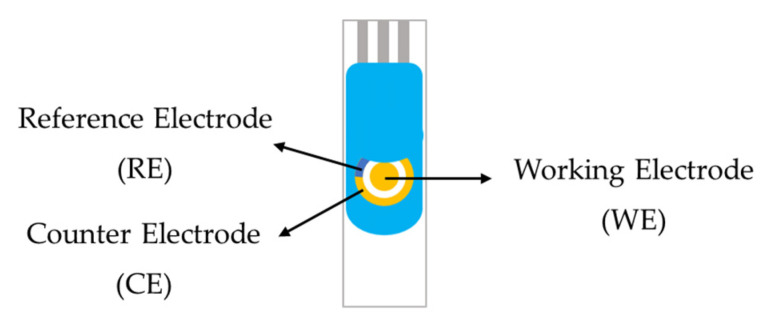
The schematic of gold screen-printed electrode.

The screen-printed electrodes (SPEs) employed in this study were obtained from Dropsens (DRP-220BT, Asturias, Spain). Each electrode was fabricated on a ceramic substrate and integrated three distinct components: a gold working electrode, a gold counter electrode, and a silver reference electrode, as illustrated in Figure 3. Gold was selected for both the working and counter electrodes due to its high conductivity, chemical stability, and biocompatibility, which are particularly advantageous for biosensing applications [41,42,43]. The silver reference electrode provided a stable potential, ensuring reproducible electrochemical measurements throughout the experiments.

On the working electrode, the sensitive layer comprising the molecularly imprinted polymer (MIP) was deposited to provide selective recognition of *Staphylococcus aureus*. This layer was tailored to enhance specificity while preserving electrode conductivity, thereby ensuring both high sensitivity and stability during analysis.

The sensor was fabricated using a composite of polymer and graphene oxide. Four different polymerization conditions were investigated by varying the monomer ratios of hydroxyproline (HYP) to methacrylamide (MAM) and hydroxyproline (HYP) to acrylamide (AAM), as summarized in Table 1. To strengthen the polymer network, 47 mg of the cross-linker N,N′-(1,2-dihydroxyethylene)bis(acrylamide) (DHEBA) was added, while 1.5 mg of azobis(isobutyronitrile) (AIBN) was incorporated as the radical initiator to initiate polymerization. All components were dissolved in 300 μL of dimethyl sulfoxide (DMSO), which served as the reaction solvent [44,45].

This condition allowed the controlled synthesis of polymer layers with different monomer ratios, enabling assessment of the optimal composition for bacterial imprinting and sensor performance.

The monomer mixture was first pre-polymerized at 70 °C on a hot plate for approximately 10 min to obtain a pre-polymer gel solution. This pre-polymer solution was then combined with graphene oxide (GO, 0.15 mg/mL) which was dispersed in deionized water by ultrasonication for 30 min at a ratio between pre-polymer and GO of 2:3 to enhance electrical conductivity [46]. A volume of 1 µL of the resulting pre- polymer/graphene oxide composite was deposited onto the working electrode surface, followed by the addition of the *S. aureus* template 1 µL which mention in Section 2.1 on top of the coating.

The modified electrodes were subsequently exposed to ultraviolet light for 3 h to facilitate drying and pre-curing of the polymer layer on the electrode surface [47]. They were then incubated in an oven at 55 °C for 15 h to complete the polymerization process. After polymerization, the bacterial template was removed by soaking the electrode in 10% acetic acid for 30 min, followed by soaking in deionized water for 30 min. The bacterial template was washed out to create specific imprinted cavities within the polymer matrix. These cavities are complementary in shape and functional groups to the target bacteria, allowing for highly selective recognition during subsequent detection.

### 2.4. Electrochemical Sensors for Analysis

Cyclic voltammetry (CV) was employed to evaluate the electrochemical response of *Staphylococcus aureus* across a range of concentrations. The fixed bacterial suspensions prepared in Section 2.1 were obtained by serial dilution in a redox couple solution consisting of potassium ferrocyanide (K_4_[Fe(CN)_6_]) and potassium ferricyanide (K_3_[Fe(CN)_6_]) mixed in a 1:1 ratio, which served as the supporting electrolyte. The final concentration range of *S. aureus* examined was 1 to 10,000 CFU/mL. For each CV measurement, 100 µL of the prepared bacterial suspension was carefully deposited onto the surface of the working electrode and allowed to interact under ambient conditions to facilitate recognition between the bacterial cells and the imprinted sites. Electrochemical measurements were then performed using a potentiostat AutoLab PGSTAT302N (Methrom Dropsens, Asturias, Spain), applying a potential sweep from −0.3 V to +0.6 V. This range was selected to capture the symmetric redox peaks of the cyclic voltammogram. The scans were carried out at a fixed rate of 50 mV/s to ensure consistent signal resolution and reproducibility. The electrochemical response was monitored approximately 2 min per cyclic voltammetry measurement.

The obtained cyclic voltammograms were analyzed to determine the electrochemical response at varying bacterial concentrations, providing the basis for evaluating the sensitivity and performance of the molecularly imprinted polymer sensor.

### 2.5. Metallurgical Optical Microscopy

Microscopic examination was carried out to observe the morphology of *S. aureus* cells as well as the cavities formed on the electrode surface after the imprinting process. A metallurgical optical microscope (BX53M, Olympus, Tokyo, Japan) was used for imaging. Observations were performed under bright-field illumination to provide clear visualization of cell structures and imprinted sites. The microscope was equipped with a 100× objective lens and a 10× ocular lens, giving a total magnification suitable for detailed surface analysis. Images were acquired systematically to document the cellular features and confirm the presence of well-defined cavities corresponding to the imprinted bacterial templates, thereby verifying the success of the molecular imprinting process.

## 3. Results

**Figure 4:**
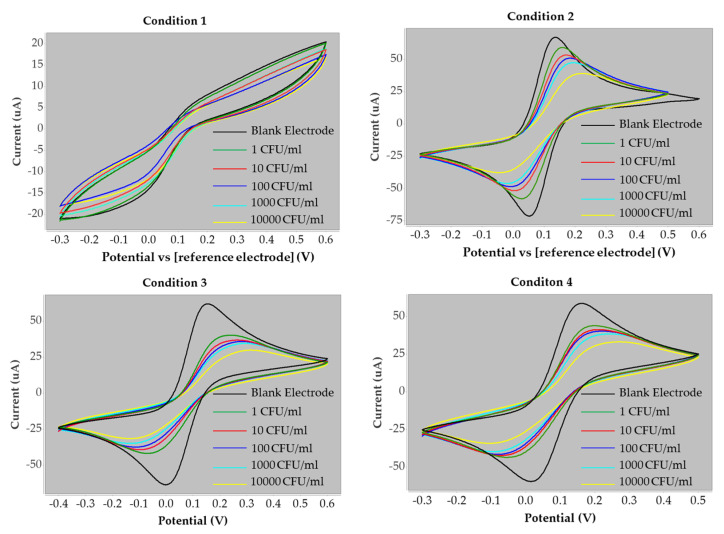
The cyclic voltammogram response of the *S. aureus* at various concentration (1–10,000 CFU/mL) obtained on the screen-printed electrode under four polymers condition. Measurements were performed at different scan rates varied from −0.3 to +0.6 V at a fixed scan rate of 50 mV s^−1^.

Cyclic voltammetry was employed to evaluate *S. aureus* at different concentrations in phosphate-buffered saline (PBS). The peak anodic current increased towards higher potentials with bacterial concentration, reflecting the onset of *S. aureus* oxidation at the working electrode surface, as illustrated in Figure 4. The current response was directly proportional to the sequential addition of *S. aureus* across the tested range of 1–10,000 CFU/mL.

In contrast, no significant variation was observed during the cathodic process, indicating that the reduction peak was unaffected by bacterial concentration. This finding suggests that the reduction current was primarily influenced by the diffusion rate of the redox couple at the electrode surface and the fraction converted to its reduced form. These results confirm that the oxidation process provides a sensitive signal for quantifying *S. aureus*, while the reduction process remains diffusion-limited and independent of bacterial concentration.

The cyclic voltammograms obtained under condition 3 were analyzed to measure both the baseline current of the blank electrode and the peak currents corresponding to each concentration of *S. aureus*. These values are summarized in Table 2. The current values reported in this study were obtained from the measurement of the oxidation peak height. To evaluate and compare the performance of the electrodes, the percentage change in current was calculated. This parameter was defined as the relative difference between the current response of the electrode in the presence of *S. aureus* and that of the blank electrode, providing a normalized measure of sensitivity across concentrations.

(2) $$\%\;\mathrm{Current}\;\mathrm{Change}=\frac{\left(I-I_{0}\right)}{I_{0}}\;\times \;100$$

where *I* is the current for concentration levels of *S. aureus* and *I*_0_ is the current of the blank electrode.

**Figure 5:**
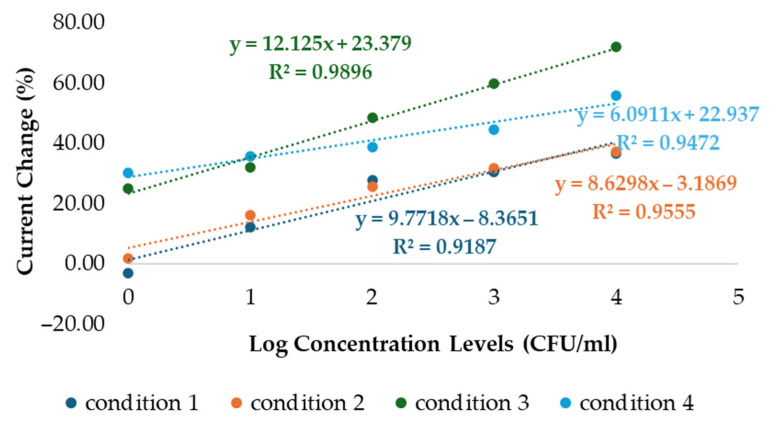
The linearity graphs of logarithmic concentration levels plot of *S. aureus* vs. the percent current change at all four polymer conditions. These results are based on experiments that were repeated three times (n = 3).

Graphs of percentage current change versus the logarithm of *S. aureus* concentration were plotted for all four polymer conditions, with the corresponding data summarized in Table 2. These plots illustrate the relationship between bacterial concentration and current response under the different synthesis conditions. A comparison of the linear responses obtained from each monomer composition is presented in Figure 5, highlighting the differences in electrode performance across the four polymer conditions.

Figure 5 illustrates the relationship between the logarithm of *S. aureus* concentration (CFU/mL, x-axis) and the corresponding current response (µA, y-axis). All four polymer conditions demonstrated positive electrochemical responses with increasing bacterial concentrations. Conditions 1 and 2, with HYP:MAM ratios of 1:2 and 2:1, showed regression coefficients (R^2^) of 0.9187 and 0.9555, respectively. However, the percentage current change observed under these conditions was lower compared to conditions 3 and 4, which employed HYP:AAM ratios of 1:2 and 2:1, yielding regression coefficients of 0.9896 and 0.9472, respectively. For conditions 1 and 2, the error bars are relatively small compared to the current values approximately ±5%. While for conditions 3 and 4, the error bars are approximately ±5–10% of the values. Overall, the error bars are not considered excessive, as the differences between conditions are still clearly distinguishable, especially for condition 3, which exhibits the highest slope (12.125) and the highest current values indicating relatively good reproducibility. These results are based on experiments that were repeated three times. Among the four conditions, condition 3 demonstrated the most favorable performance, providing a stronger positive response at higher bacterial concentrations.

**Figure 6:**
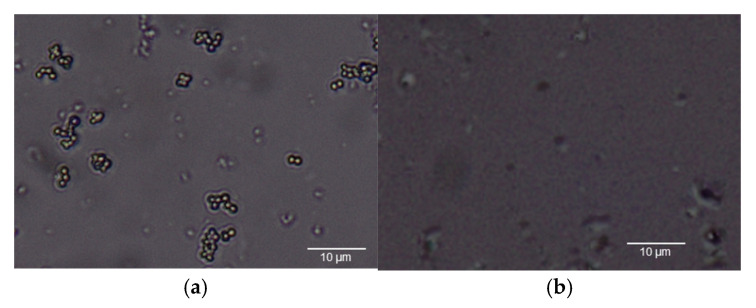
(**a**) The morphology of *S. aureus* and (**b**) cavities of *S. aureus* after wash out template.

To further validate the imprinting process, metallurgical optical microscopy was employed to visualize the electrode surface. Figure 6a shows the morphology of intact *S. aureus* cells prior to template removal, while Figure 6b depicts the electrode surface after bacterial extraction using 10% acetic acid and deionized water. Distinct cavities corresponding to the imprinted bacteria were observed, maintaining the original cellular morphology with an average diameter of approximately 0.8 µm. These microscopic findings are consistent with the superior electrochemical response observed under condition 3, confirming that this polymer condition produced well-defined and highly effective imprinting sites for *S. aureus* recognition.

**Figure 7:**
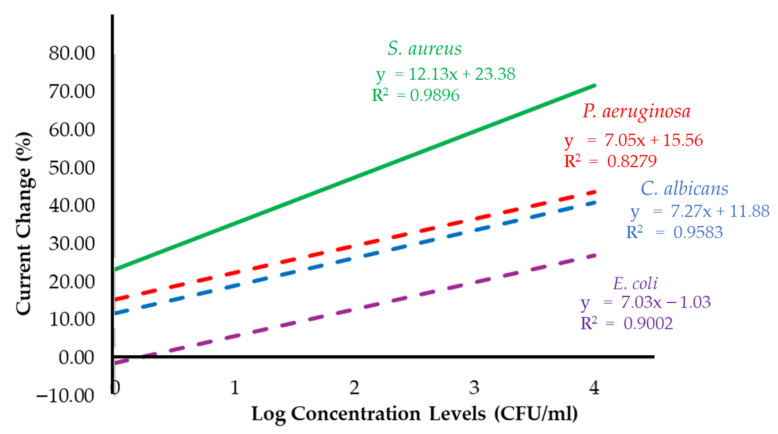
The percent current change as a linear function of log concentration levels of *S. aureus* in condition 3 compared with the results from the negative control bacteria experiments by using *C. albicans*, *E. coli* and *P. aeruginosa*.

For the selectivity test, *Candida albicans* (*C. albicans*) was selected as a control organism because, like *S. aureus*, it has a similar cell diameter. In addition, *Escherichia coli* (*E. coli*) and *Pseudomonas aeruginosa* (*P. aeruginosa*) were included as non-target bacteria, representing Gram-negative bacteria with distinct morphological differences from *S. aureus*. The electrochemical responses of these non-target organisms were measured to assess the sensor’s specificity, and the results are summarized in Table 3. These comparisons confirm that the sensor can reliably distinguish *S. aureus* from other microorganisms, demonstrating strong selectivity and potential for practical diagnostic applications (Figure 7).

The negative control organisms produced only minor changes in current compared with the strong responses obtained for *S. aureus* across the concentration range of 1–10,000 CFU/mL. The biosensor incorporating a gold electrode demonstrated the highest sensitivity toward *S. aureus*, with a value of 12.13 (% current change per log concentration). In comparison, the calculated sensitivities for *C. albicans*, *E. coli*, and *P. aeruginosa* were 7.27, 7.03, and 7.05 (% current change per log concentration), respectively. These results confirm the superior responsiveness of the sensor to *S. aureus* relative to non-target organisms, emphasizing its strong selectivity and potential diagnostic utility. The limit of detection (LOD) for *S. aureus* was calculated from the calibration curve obtained by linear regression between the log concentration (CFU/mL) and the electrochemical response (% current change, µA), using the standard formula [48,49]:

LOD = 3.3(σ/S)(3)

From the results, the slope and standard deviation were 12.12 and 0.05, respectively, yielding a calculated LOD of 0.014 in log scale. This corresponds to an actual concentration of 1.031 CFU/mL after back-transformation. These findings ensure that the biosensor can reliably detect *S. aureus* even at very low bacterial counts. Demonstrating strong selectivity and potential diagnostic utility. Together, these findings reinforce the ability of the biosensor to provide accurate and reliable detection of *S. aureus*, even in the presence of non-target microorganisms with similar size or differing morphology.

## 4. Discussion

In this study, four different polymer conditions were evaluated to optimize the biosensor for *S. aureus* detection. Among these, condition 3 (HYP:AAM = 1:2) demonstrated the most effective performance. The cyclic voltammograms obtained from the screen-printed electrodes confirmed the ability of this condition to distinguish signals across different *S. aureus* concentrations, validating the success of the imprinting process.

Interestingly, a decrease in the percentage of current change was observed with increasing bacterial concentration. This trend suggests that as more *S. aureus* cells bound to the imprinted sites on the electrode surface, the biosensor experienced greater impedance, thereby limiting electron transfer. Such behavior is consistent with the expected blocking effect caused by bacterial adhesion, confirming that the electrode surface was effectively imprinted for selective recognition [50]. The selectivity of the sensor was further assessed by testing against non-target organisms, including *C. albicans*, *E. coli*, and *P. aeruginosa*. Compared with *S. aureus*, these control organisms produced significantly lower current changes, and no substantial variation was observed across their concentration ranges. These findings reinforce the specificity of the polymer formulation under condition 3, demonstrating that the sensor can reliably differentiate S. *aureus* from other microorganisms with similar size or distinct morphology [51,52].

In addition, these results highlight the potential of the developed biosensor as a practical tool for rapid and selective detection of *S. aureus*, offering promising applications in clinical diagnostics and infection monitoring.

## 5. Conclusions

This research focused on the development of a biosensor based on the molecularly imprinted polymer (MIP) technique for the rapid detection of *Staphylococcus aureus*. The system employed electrochemical analysis using cyclic voltammetry, which enabled sensitive and selective recognition of bacterial cells. Among the four polymer conditions tested, the composition with a hydroxyproline (HYP) to acrylamide (AAM) ratio of 1:2 provided the best performance. This condition demonstrated the highest sensitivity, with a response of 12.13 (% current change per log concentration), and the strongest linearity, with a regression coefficient (R^2^) of 0.9896. The calculated limit of detection was as low as 1.031 CFU/mL, confirming the sensor’s ability to detect very low bacterial concentrations. Specificity testing against *Candida albicans*, *Escherichia coli*, and *Pseudomonas aeruginosa* further validated the selectivity of the sensor, as none of the negative control organisms produced current responses comparable to *S. aureus*. These findings highlight the reliability of the developed biosensor in distinguishing the target pathogen from other microorganisms.

Overall, this research demonstrates the potential of a MIP-based electrochemical biosensor as a rapid, sensitive, and selective diagnostic tool for *S. aureus*. Future development should focus on validating the system with real clinical samples to advance its application in hospital diagnostics and patient care.

## Figures and Tables

**Table 1:** The amounts of monomers in various conditions.

Condition	Ratio (n:n)	Ratio (mmol:mmol)	HYP (mg)	MAM (mg)	AAM (mg)
1	1:2	0.1:0.2	13.1	17.0	-
2	2:1	0.2:0.1	26.2	8.5	-
3	1:2	0.1:0.2	13.1	-	14.2
4	2:1	0.2:0.1	26.2	-	7.1

**Table 2:** The current data information at all four polymer conditions: Condition 1 (HYP: MAM = 1:2), Condition 2 (HYP: MAM = 2:1), Condition 3 (HYP: AAM = 1:2), and Condition 4 (HYP: AAM = 2:1).

**Condition 1**	**Current (** **µA)**	**∆I (µA)**	**Current change (%)**
Blank	4.82		
1 CFU/mL	4.96	−0.14	−2.97
10 CFU/mL	4.23	0.59	12.30
100 CFU/mL	3.47	1.35	27.97
1000 CFU/mL	3.34	1.49	30.81
10,000 CFU/mL	3.05	1.77	36.64
**Condition 2**	**Current (** **µA)**	**∆I (µA)**	**Current change (%)**
Blank	71.85		
1 CFU/mL	70.43	1.42	1.97
10 CFU/mL	60.09	11.76	16.37
100 CFU/mL	53.22	18.63	25.93
1000 CFU/mL	48.96	22.90	31.87
10,000 CFU/mL	45.00	26.85	37.37
**Condition 3**	**Current (** **µA)**	**∆I (µA)**	**Current change (%)**
Blank	82.38		
1 CFU/mL	61.54	20.84	25.30
10 CFU/mL	55.83	26.55	32.23
100 CFU/mL	42.31	40.07	48.64
1000 CFU/mL	33.06	49.32	59.87
10,000 CFU/mL	22.98	59.40	72.10
**Condition 4**	**Current (** **µA)**	**∆I (µA)**	**Current change (%)**
Blank	57.95		
1 CFU/mL	40.43	17.52	30.23
10 CFU/mL	37.19	20.76	35.82
100 CFU/mL	35.36	22.59	38.99
1000 CFU/mL	31.97	25.98	44.84
10,000 CFU/mL	25.40	32.55	56.18

**Table 3:** The current data information of negative control bacteria.

***C. albicans***	**Current (** **µA)**	**∆I (µA)**	**Current change (%)**
Blank	2.11		
1 CFU/mL	1.89	0.22	10.33
10 CFU/mL	1.70	0.41	19.43
100 CFU/mL	1.47	0.64	30.28
1000 CFU/mL	1.45	0.66	31.33
10,000 CFU/mL	1.25	0.86	40.76
***E. coli***	**Current (µA)**	**∆I (µA)**	**Current change (%)**
Blank	2.63		
1 CFU/mL	2.58	0.05	1.86
10 CFU/mL	2.47	0.16	6.16
100 CFU/mL	2.45	0.18	6.84
1000 CFU/mL	2.10	0.53	20.30
10,000 CFU/mL	1.84	0.79	29.92
***P. aeruginosa***	**Current (µA)**	**∆I (µA)**	**Current change (%)**
Blank	2.59		
1 CFU/mL	2.35	0.24	9.31
10 CFU/mL	1.84	0.75	28.96
100 CFU/mL	1.77	0.82	31.81
1000 CFU/mL	1.59	1.00	38.57
10,000 CFU/mL	1.56	1.03	39.77

## Data Availability

The original contributions presented in this study are included in the article. Further inquiries can be directed to the corresponding author.
